# Bio-loggers and miRNAs are innovative tools for measuring physiological changes in lambs during transport

**DOI:** 10.1093/jas/skaf123

**Published:** 2025-04-17

**Authors:** Isabella Manenti, Irene Viola, Francisco Canto, Paolo Accornero, Paola Toschi, Carmine Versace, Elisabetta Macchi, Eugenio Martignani, José Alfonso Abecia, Silvia Miretti

**Affiliations:** Department of Veterinary Sciences, University of Turin, Grugliasco, Italy; Department of Veterinary Sciences, University of Turin, Grugliasco, Italy; Instituto de Investigaciones Agropecuaria, Santiago, Chile; Department of Veterinary Sciences, University of Turin, Grugliasco, Italy; Department of Veterinary Sciences, University of Turin, Grugliasco, Italy; Department of Veterinary Sciences, University of Turin, Grugliasco, Italy; Department of Veterinary Sciences, University of Turin, Grugliasco, Italy; Department of Veterinary Sciences, University of Turin, Grugliasco, Italy; IUCA, Faculty of Veterinary Medicine, University of Zaragoza, Zaragoza, Spain; Department of Veterinary Sciences, University of Turin, Grugliasco, Italy

**Keywords:** adaptation response, microRNA, transport, sensors, sheep

## Abstract

For livestock, transport can involve several potential stressors including human handling, stocking density, air temperature, noise, vibration, and loading/unloading procedures. The animal’s perception of and its ability to adapt to those stimuli are not fully understood, which makes it difficult to carry out welfare assessments. This study aimed to detect stressful moments in lambs during transport through changes in physiological and molecular markers. Data obtained from bio-loggers that record physiological variables and an evaluation of molecular biomarkers such as cortisol and circulating microRNAs (c-miRNAs) identified the most stressful moments of transport, which can be a valuable tool for evaluating and improving transport conditions for livestock. Rasa Aragonesa lambs were implanted with bio-loggers that record subcutaneous body temperature (**BT**) and heart rate (**HR**). Plasma and saliva were sampled for molecular analyses; specifically, saliva for cortisol concentrations, plasma for creatine kinase (**CK**), and lactate dehydrogenase (**LDH**), and plasma and saliva for c-miRNAs expression profiles. Immediately after the lambs were unloaded, the bio-loggers registered a significant (*P* < 0.05) spike-in HR and a drop in BT, and salivary cortisol concentrations increased significantly (*P *< 0.05), which indicated unloading as one of the main stressful points for the lambs. Out of the 17 miRNAs tested, 4 showed a significant difference in expression (*P *< 0.05). MiR-23a and -27a were both expressed in plasma and saliva, while miR-17 and -24 were most expressed in saliva after unloading. Finally, the expression of plasmatic miR-23a, -24, and 27a were significantly (*P *< 0.05) positively correlated with the LDH whose concentrations together with those of CK are significantly increased (*P *< 0.01) after unloading. The study identified the timing of a lamb’s adaptation response during and after transport, which reflected the dynamic nature of ovine plasma and salivary miRNAs during transport-induced stress, giving them the potential to be biomarkers that can be useful in animal welfare assessments.

## Teaser

This research introduces innovative approaches to measure adaptation response in lambs during transport, offering valuable tools for enhancing animal welfare assessment. Through the integration of advanced technologies and biomarkers, this work proposes non-invasive methods to trace how lambs answer to stressful transportation stimuli. The results could be useful to revise or rethink management practices in animal transport.

## Introduction

Livestock transport is an animal welfare issue, but an essential activity in the meat production system.

The impact of animal welfare has a direct effect on meat quality and, before that, on the mental and physical state of the animals, which has a significant effect on the profitability of the food chain industry, and is an ethical topic that has become important to consumers (Tamioso et al., 2018). Recently, the World Organization for Animal Health (**WOAH**) has improved the international science-based standards that encourage measures that protect food-producing animals that are kept for farming purposes (WOAH, 2022) during transport and related operations. Transportation is one of the most stressful conditions to which a farm animal can be exposed and, because it usually occurs close to the time of slaughter, it can have a significant effect on the quality of the meat (De la Fuente et al., 2010; Zhong et al., 2011). During transport, animals can be exposed to a variety of environmental stressors, including changes in ambient temperature, vibration, noise, stocking density, and human handling, which are some of the most common physical and psychological discomforts (Cockram et al., 1996; Pascual-Alonso et al., 2017; Nielsen et al., 2022). Those stressors perturb homeostasis and an adaptive response mediated by both the sympathetic adrenal medullary (**SAM**) and the hypothalamic–pituitary–adrenal (**HPA**) axes is activated to restore balance, and physiological responses associated with reactivity (heart rate [**HR**] and respiratory frequency) and release of hormones are triggered, which are followed by behavioral changes. In several previous studies, the impact of loading, transport, and unloading on the adaptive response of lambs was investigated and in part the time to regain homeostatic balance too (Miranda-de la Lama et al., 2018; Carnovale et al., 2021; Vogel et al., 2024).

To date, innovative approaches to quantifying the welfare state of food-producing animals have been closely associated with the digital revolution in agriculture (Precision Livestock Farming, PLF), which has aided in quantifying animal welfare based on variables recorded by sensors. Advances in engineering and reductions in costs of the new electronic technologies have led to the development of many sensor-based solutions for the livestock industry (Caja et al., 2016; Halachmi et al., 2019). Those sensors can collect data automatically and in real-time, which allows for the early detection of specific problems (e.g., threats to production, health, and welfare) at the group or individual level (Caja et al., 2016; Maltz, 2020).

In recent years, the development of bio-loggers for monitoring, for example, BT, respiration, and HR, or the activity of the animals, have helped in understanding how environmental factors (building and management) affect an animal’s resiliency to stressors (Pascual-Alonso et al., 2017; Chmura et al., 2018; Forin-Wiart et al., 2019).

On the other hand, molecular biology has identified molecules that, combined with other parameters, can act as biomarkers for the welfare state of livestock. Stress stimuli promptly activate the SAM and HPA axes, which influence behavior, physiological factors, and molecular expression, such as the microRNAs (miRNAs) (Chen et al., 2015). MiRNAs are small non-coding RNA molecules that are involved in a wide range of biological processes through posttranscriptional gene expression regulation and are important in regulating hormone and immune responses. An interesting aspect in identifying miRNA molecules that can act as biomarkers is their highly regulated spatial and temporal expression, secretion from cells into extracellular compartments, and stability in body fluids (c-miRNAs). For those reasons, c-miRNAs are among the most promising clinical biomarkers for the diagnosis of a variety of diseases and stress disorders in farm animals (Dong et al., 2017; Miretti et al., 2020; Do et al., 2021).

At present, there is still a lack of knowledge about the possible effects of transport on c-miRNAs pattern involved in the lambs’ adaptation response. Assessing animal welfare in an objective way requires measuring parameters in a quantifiable and reproducible manner and bio-loggers and microRNAs could be promising tools for this purpose.

On the basis of this evidence, we hypothesized that lambs would show a change in subcutaneous BT and HR at the time of activation of the adaptive response during transport, allowing us to detect and study the expression pattern of c-miRNAs at the same time points. We also hypothesized that basal expression conditions would be restored a few hours after the stress stimuli. To verify this hypothesis, in this study, a short-road transportation and related procedures were selected as stress models. Body temperature (**BT**), HR, and saliva cortisol concentrations were the physiological indicators that were used to verify the activation of the adaptation response axes. The study evaluated the effects of transport on the adaptive response and welfare status of Rasa Aragonesa lambs as documented by new technologies (physio-bio-loggers) and innovative molecular markers (c-miRNAs).

## Material and Methods

### Experimental design

The study was conducted at the experimental farm of the University of Zaragoza, Spain (41° 63 N), following procedures approved by the Ethics Committee for Animal Experiments at the University of Zaragoza (PI29/21). The care and use of animals followed the Spanish Policy for Animal Protection (RD 53/2013), which meets the European Union Directive 2010/63 on the protection of animals used for experimental and other scientific purposes.

**Figure 1.**
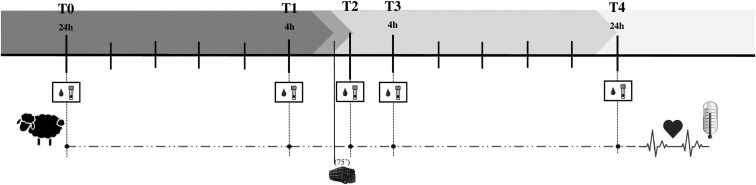
Overview of experimental design. Saliva and plasma samples were collected from Rasa Aragonesa lambs 5 times: twice before transport (T0 and T1: 24 h and 4 h PRE, respectively), once immediately after a 75-min transport (T2) and twice after transport (T3 and T4: 24 h and 4 h POST, respectively). Implanted bio-loggers recorded HR and BT throughout the study.

The types and timing of sample collection are summarized in Fig. 1. HR and BT were recorded by subcutaneous implanted bio-loggers, which began the day before transport and up to 24 h after transport. Blood and saliva were collected 24 h and 4 h before the onset of transport (T0 to T1), and immediately after the unloading of the lorry at the end of the transport (T2), and 4 h and 24 h after the transport (T3 to T4). The transport lasted 75 min.

### Lambs and bio-logger implantation

Fourteen (8 males, 6 females) Rasa Aragonesa lambs (mean age [±SD]: 94.4 ± 2.2 d) that had been born within a week in early Feb, were selected at the end of the fattening period (mean live weight: 23.9 ± 2.3 kg), and received a surgically implanted subcutaneous temperature (BT) and HR bio-logger (8.3 × 25.4 mm and 3.3 g; DST micro-HRT, Star Oddi, Iceland) 7 d before transport day. Bio-loggers were sterilized by immersion in 0.55% ortho-phthalaldehyde (CIDEX-OPA solution, Johnson & Johnson, New Jersey, USA) for 24 h. Lambs were placed in a cradle in dorsal recumbency. A povidone–iodine soap solution (Betadine Scrub 7.5%, Alcon Laboratories, Inc., Fort Worth, TX) was used to prepare the skin for surgery, and 1 ml of local anesthetic (lidocaine hydrochloride, Anesvet, Ovejero, León, Spain) was injected. The bio-logger was placed subcutaneously on the left thorax, directly above the heart, with the sensor axis parallel to the heart axis (Abecia et al., 2021). An incision was made in the skin and a pocket was created to hold the bio-logger. To secure the bio-logger in the skin pocket, a 2/0 absorbable suture (Novosyn, B-Braun, Melsungen, Germany) was threaded through a small hole at the tip of the bio-logger. The incision was closed with 2-3 sutures, and the scar was sprayed with aluminum (Aluspray, Vetoquinol, Madrid, Spain). A similar surgical procedure was used to remove the bio-logger at the end of the sampling period (Abecia et al., 2021). Animals were housed in a 25-m^2^ paddock and were fed a 14.8% crude protein concentrate (Cadecor-2, Agroveco, Zaragoza, Spain), and barley straw and water available *ad libitum*. Ambient temperature (*T*, °C) and relative humidity (**RH**, %) on the farms and in the lorry were recorded by mini data loggers (Testo 174H, Testo SE & Co. KGaA, Titisee-Neustadt, Germany).

### BT and HR analyses

The bio-loggers were programmed to record BT and HR data at 10-min intervals on the day before transport and up to 6 d after transport, and at 1-min intervals on the day of transport. The use of those devices in sheep studies has been validated (Fuchs et al., 2019; Abecia et al., 2021, 2025). After 11 d, the bio-loggers were retrieved and the data were downloaded through a communication box and the Mercury software v5.83 (Star Oddi, Gardabaer, Iceland).

The bio-logger records HR by a leadless single-channel electrocardiogram (ECG), which records burst measurements at set time intervals and calculates the mean HR for each record (Abecia et al., 2021). Each burst is assigned a quality index (**QI**) for validation, which is calculated by the application software. The algorithm calculates the QI in a 2-step process; initially, it searches the record for QRS waves and calculates each R-R interval. If there is <20% variability within the R-R intervals, the grade is set to QI = 0 (good). If there is no R-R interval, or HR is above or below a certain defined threshold, QI = 3 (poor). In the second step, the lower-level threshold (**LLT**) and the higher-level threshold (**HLT**) are identified. If the thresholds overlap (LLT ≥ HLT), all potential R waves are of a similar grade, which is used to calculate beats per minute (bpm), and the QI is set to 1 (good). Otherwise, potential R-waves graded above the HLT are used in the calculation, and the QI is still set to 1 (good), except if 1 or more potential R-waves are graded between the LLT and HLT. In that case, the rating is considered somewhat ambiguous, and the QI is set to 2 (fair). If only a single R-wave is above the HLT, the QI is set to 3 (poor). In all cases, HR that graded QI = 2 or 3 were excluded from the analyses because they cannot be considered reliable (Star-Oddi, 2024).

### Transport

In the first week of May, the lambs were transported 73 km (mean speed 68 km/h) in the central part of the lower deck of the lorry, in a journey that began and ended at the experimental farm of the University of Zaragoza. Including loading and unloading, the journey lasted 2.5 h. Lambs weren’t feed-deprived before transport. The livestock lorry (IVECO, Turin, Italy; model ML80E14, 8,000 kg capacity), which was designed for sheep transport, had 2 axles and 2 floors, and 5 m × 2.25 m × 2.20 m box. Lambs were not divided and were placed as a group during transport. The vehicle had a hydraulic elevator for loading and unloading. Space allowance was 0.32 m^2^ per lamb (Consortium of the Animal Transport Guides Project, 2018). Environmental temperature and relative humidity (average during transport = 19.91 °C and 38.74%, respectively) were recorded every 5 min by data loggers (Testo) that had been positioned at lamb height. The transport route consisted of a well-maintained 2-lane highway with a high-quality road surface, free of potholes or irregularities that could impact travel conditions.

### Saliva and blood collection

Blood and saliva samples were collected from lambs 24 h and 4 h before the onset of the transport (PRE: T0 and T1), immediately after the unloading of the lorry at the end of the transport (T2), and 4 h and 24 h after the transport (POST: T3 and T4). Blood was collected from the jugular vein by 10-mL VACUETTE Tubes K3E K3EDTA with a VACUETTE Multiple Use Drawing Needle 20Gx1½” (Greiner Bio-One GmbH, Kremsmünster, Austria). Plasma was separated by centrifugation at 3,500 rpm for 10 min at 5 °C, aliquoted in aliquots of 1.3 mL, and stored at −80 °C. Saliva samples were collected with a polyethylene pad (Salivette, Sarsted AG & Co., Nümbrecht, Germany). A clamp was used to insert the swab into the animal’s mouth, which remained there for 2 min to allow the lamb to chew it and the swab to absorb saliva. The saliva was collected from the swab after sampling by centrifugation at 3,500 rpm for 10 min at 5 °C and stored at −80 °C (Gröschl and Rauh, 2006; Nater et al., 2006; Topkas et al., 2012).

### Estimation of salivary cortisol

To measure the concentration of saliva cortisol, an ELISA immunoassay was performed by a commercial DRG Salivary Cortisol ELISA Kit (DRG Instruments GmbH, Germany), following the manufacturer’s protocol. The sensitivity range of the assay was between 0.09 and 30 ng/mL. According to the manufacturer, the kit produces the following cross-reactivities: 23.40% with progesterone, 11.39% with DHEA, 1.97% with androstenedione, and 1.47% with estriol. The results are expressed as the concentration of cortisol in saliva (ng/mL).

### Extraction and analysis of saliva and plasma miRNAs

Before extraction, samples of plasma and saliva were each centrifuged at 15,000 rpm for 5 min at room temperature. Extraction was performed using 500 μL of centrifuged plasma or saliva by a Maxwell RSC miRNA Plasma or Serum kit (Promega, Madison, Wisconsin, United States) following the manufacturer’s protocol. To evaluate the quality of the extraction, 1 μL of UniSp2,4,5 spike-in (Qiagen, Hilden, Germany) was added to each sample. miRNA quantification was performed by Qubit microRNA Assay kit and the Quantus Fluorometer (Invitrogen, Thermo Fisher Scientific Inc., Waltham, MA, United States), following the manufacturer’s guidelines. The miRNA samples were stored at −80 °C.

Immediately after extraction and quantification, 0.8 μL of microRNA samples were retrotranscribed in cDNA by a miRCURY LNA RT Kit (Qiagen) in a final volume of 10 μL, following the manufacturer’s instructions. At that stage, for the evaluation of the quality of the reverse transcription, 0.5 μL of UniSp6 spike-in (Qiagen) was added to each sample. The cDNA samples were stored at −20 °C.

For Real-Time PCR analyses, the cDNA was diluted 30-fold before use, and 3 μL were assayed with 7 μL of PCR mix following the protocol for the miRCURY LNA SYBR Green PCR Kit (Qiagen).

### Creatine kinase and lactate dehydrogenase (LDH) quantifications

A computer-controlled automatic analyzer for clinical chemistry, BT 3500 Random Access Chemistry Analyzer (Biotecnica Instruments, Rome, Italy) was used to quantify the creatine kinase (CK) (CK NAC – catalog no. 123L, Biotecnica Instruments, Rome, Italy) and LDH (LDH-L – catalog no. 274L, Biotecnica Instruments, Rome, Italy) enzyme concentrations. Both enzymes’ analyses are based on an optimized UV test according to DGKC (German /IFCC (International Federation of Clinical Chemistry and Laboratory Medicine). The diagnostic reagents used were provided as ready for use for quantitative in vitro determination of CK and LDH in serum or plasma on photometric systems. The limits of enzymes’ quantification were 12 up to 1,300 U/L and up to 1,200 IU/L for CK and LDH respectively. Manufacturer’s control solutions for internal quality control were used.

For each lamb, 200 μL of the stored −20 °C plasma samples were analyzed. CK and LDH concentrations were measured before transportation (PRE: T0 to T1), immediately after unloading (T2), 4 h (T3), and 24 h (T4) after the trip.

### Statistical analyses

The day of transport (duration = 75 min) was divided into 7 phases (2 h before transport, loading, lambs loaded in the stopped lorry, transport, unloading, first hour after transport, and second hour after transport). Mean (±SE) BT and HR in each phase were calculated and differences were assessed statistically by an ANOVA test for repeated measures and Tukey test.

For the statistical analyses of salivary cortisol concentrations, BT, HR, CK, and LDH levels, and the differential expression of miRNAs, the T0 and T1 samples were used to provide baseline concentrations. A 1-way ANOVA test for repeated measures (if data were normally distributed) or a Friedman’s test and a Mann–Whitney *U* test (if data not normally distributed) in GraphPad Prism 9 for Windows, version 9.0.0, GraphPad Software, LCC were used for these analyses. The level of statistical significance was set to *P *< 0.05. For the miRNAs, the statistical analyses were performed on ∆ Cq, which was the difference between the Cq of each sample and the Cq of the normalizer Unisp6. For that reason, the higher the ∆ Cq, the less the miRNA is expressed. To assess the significance of the correlations between the miRNA expression profiles and molecular variables (cortisol, CK, and LDH), Pearson correlation tests were used.

## Results

### Handling and transport events affected subcutaneous BT and HR

**Figure 2.**
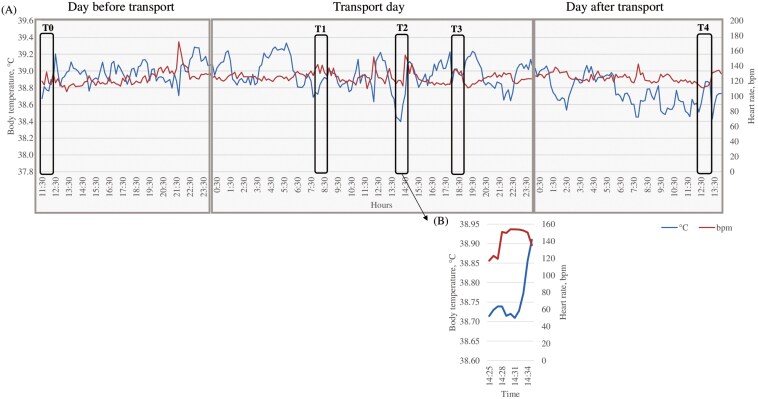
Overview of bio-logger data collected in the experiment. The bio-loggers were programmed to record lamb BT and HR at 10-min intervals on the day before transport and up to 6 d after transport, and at 1-min intervals on the day of transport (75 min duration). The hourly average of BT and HR is presented in A. Details of the unloading of the animals (T2) are presented in B. T0 (24 h before transport), BT = 39.20 ± 0.35 °C and HR = 117.65 ± 11.74 bpm; T1 (4 h before transport), BT = 38.86 ± 0.30 °C and HR = 130.06 ± 19.53 bpm; T2 (immediately after transport), BT = 38.73 ± 0.76 °C and HR = 154.07 ± 34.86 bpm; T3 (4 h after transport), BT = 39.18 ± 0.51 °C and HR = 114.44 ± 14.85 bpm; T4 (24 h after transport) BT = 38.87 ± 0.32 °C and HR = 116.56 ± 14.78 bpm.

Sensor data from the 48-h sampling period is shown in Fig. 2. At T0 (24 h before transport) the average BT of the lambs was 39.20 ± 0.35 °C, and HR was 117.65 ± 11.74 bpm. At T1 (4 h before transport), average BT was 38.86 ± 0.30 °C and HR was 130.06 ± 19.53 bpm. At T2 (immediately after transport), average BT was reduced (38.73 ± 0.76 °C) and HR was elevated (154.07 ± 34.86 bpm). At T3 and T4 (4 h and 24 h after transport), average BT was 39.18 ± 0.51 °C and 38.87 ± 0.32 °C, and HR was 114.44 ± 14.85 bpm and 116.56 ± 14.78 rpm, respectively.

### Salivary cortisol and bio-logger data analyses identify unloading as the most stressful stage

**Figure 3.**
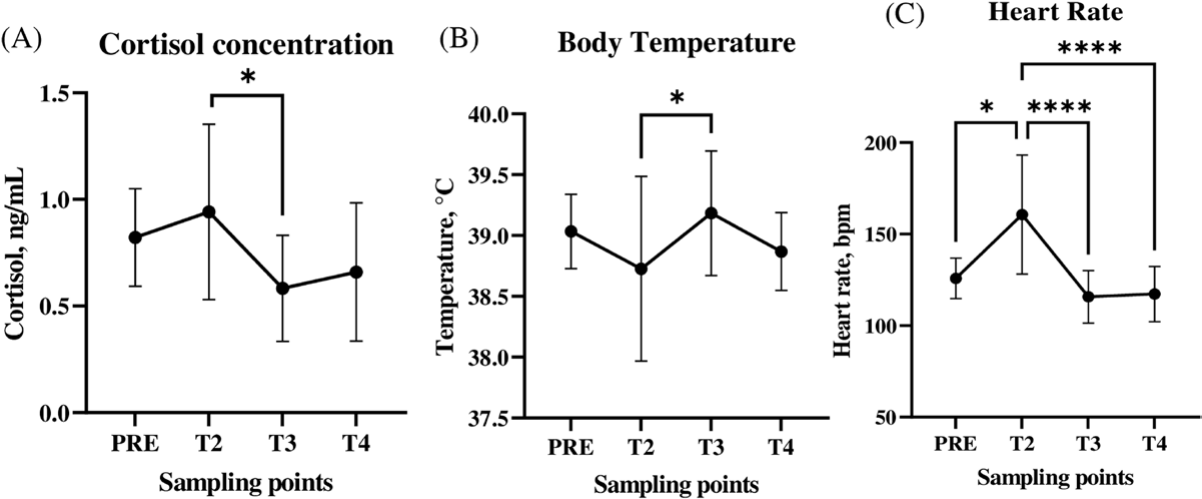
Physiological parameters. Average salivary cortisol concentration (A), BT (B) and HR (C) of Rasa Aragonesa lambs at 5 sampling points (PRE: average of T0 and T1). Values are expressed ± SD. *~ *P *< 0.05; **** ~ *P *< 0.0001.

Salivary cortisol concentrations ranged between 0.15 and 2.1 ng/mL and the average (± SD) was 0.8 ± 0.36 ng/mL. Accordingly to no statistical differences in salivary cortisol concentrations between males and females, the gender didn’t influence the results. Based on the experimental design time points, the highest cortisol concentration was immediately after the animals unloaded (T2) (0.94 ± 0.40) ng/mL, which was significantly (*P *= 0.01) higher than they were at T3, when they were lowest (mean = 0.56 ± 0.24 ng/mL) (Fig. 3A).

In the experiment, BT ranged between 36.99 °C and 39.96 °C and the average was 38.97 ± 0.52 °C. BT immediately after unloading (T2) was significantly (*P *< 0.05) lower (38.75 ± 0.05 °C) than it was 4 h posttransport (T3) (39.18 ± 0.50 °C), when average BT was highest (Fig. 3B). HR ranged from 93 to 246 bpm and was significantly higher after the unloading procedure (T2) (average = 154.07 ± 34.86 bpm), which was higher than it was at any other stage (Fig. 3C). From these results, it could be supposed that the unloading procedure was the most stressful event in the transport process.

### Changes in miRNA expression in plasma and saliva parallel the adaptation response

From published studies, 17 miRNAs (Table 1) among those well detectable in saliva (Patel et al., 2011) and/or linked to stress-response and animal welfare (Jin et al., 2016; Wiegand et al., 2018; Lee et al., 2019; Guelfi et al., 2022), were selected and were evaluated based on a representative number of saliva and plasma samples (2 lambs for 2 sampling points for each miRNA). Of those, 7 miRNAs (miR-17, -19b, -23a, -24, -27a, -143, and -191) were sufficiently expressed, especially in saliva, in the 2 matrices, which were evaluated in all samples.

**Table 1.** Designation of microRNAs

miRNA name	Primer ID	Qiagen GeneGlobe ID
miR-16b	oar-miR-16b	YP02115941
miR-17	oar-miR-17-5p	YP02110961
miR-19b	hsa-miR-19b-3p	YP00204450
miR-21	oar-miR-21	YP02110192
miR-23a	oar-miR-23a	YP02103289
miR-24	hsa-miR-24-3p	YP00204260
miR-26a	hsa-miR-26a-5p	YP00206023
miR-27a	hsa-miR-27a-3p	YP00206038
miR-29a	oar-miR-29a	YP02105177
miR-30c	oar-miR-30c	YP02114922
miR-99a	bta-miR-99a-5p	YP00205945
miR-106a	oar-miR-106a	YP02110125
miR-126a	hsa-miR-126-5p	YP00206010
miR-143	hsa-miR-143-3p	YP00205992
miR-191	cfa-miR-191	YP00205972
miR-221	bta-miR-221	YP02111922
miR-223	hsa-miR-223-3p	YP00205986

Abbreviations: miR, microRNA; oar, ovis aries; bta, bos taurus; has, homo sapiens; cfa, canis lupus familiaris.

**Figure 4.**
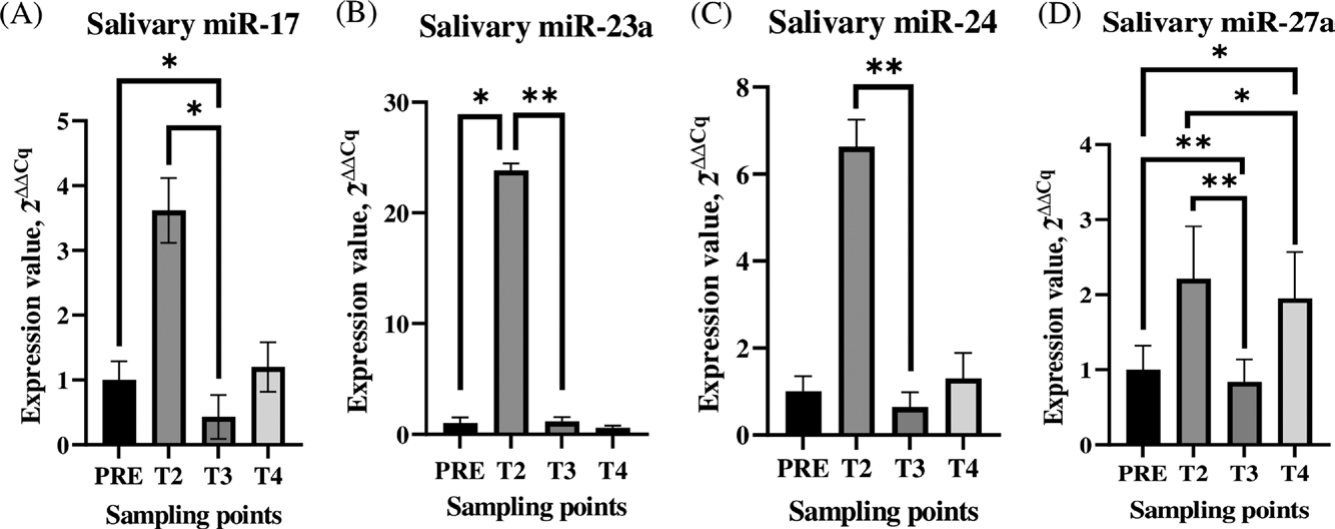
Statistically significant miRNAs in saliva. Statistically significant miRNA expression profiles in the saliva of Rasa Aragonesa lambs at 5 sampling points (PRE: average of T0 and T1). For clarity, miRNAs expression profiles are expressed in 2^−∆∆Cq^. * ~ *P *< 0.05; ** ~ *P *< 0.01.

Among those, significant differences in expression were exhibited by 4 miRNAs (miR-17, -23a, -24, and -27a) in saliva and 2 miRNAs (miR-23a and -27a) in plasma. For all of those miRNAs, expressions were significantly (*P *< 0.05) higher after unloading (T2) than they were at the next sampling point (T3). In saliva, the ∆Cq of miR-17 at T2 was 15.35 ± 1.57 and, at T3, was 18.46 ± 0.97. Furthermore, that miRNA was significantly (*P *< 0.05) less expressed at T3 than it was at basal sampling PRE (T0 and T1) (16.68 ± 0.91) (Fig. 4A). The ∆ Cq of miR-23a at T2 was 12.93 ± 1.77, which was significantly more expressed than it was at PRE (16.50 ± 1.50) (*P *< 0.05) and at T3 (17.44 ± 1.16) (*P *< 0.01) (Fig. 4B). The ∆ Cq of miR-24 was significantly (*P *< 0.01) more expressed at T2 (14.58 ± 1.97) than it was at T3 (17.91 ± 1.07) (Fig. 4C). In saliva, the expression of miR-27a was significantly higher at T2 (12.98 ± 2.21) than it was at T3 (17.08 ± 0.84) (*P *< 0.01) or T4 (16.44 ± 1.95) (*P *< 0.05). In addition, the basal expression of miR-27a in saliva (PRE; 14.55 ± 1.00) was significantly higher than it was at T3 (*P *< 0.01) or T4 (*P *< 0.05) (Fig. 4D).

**Figure 5.**
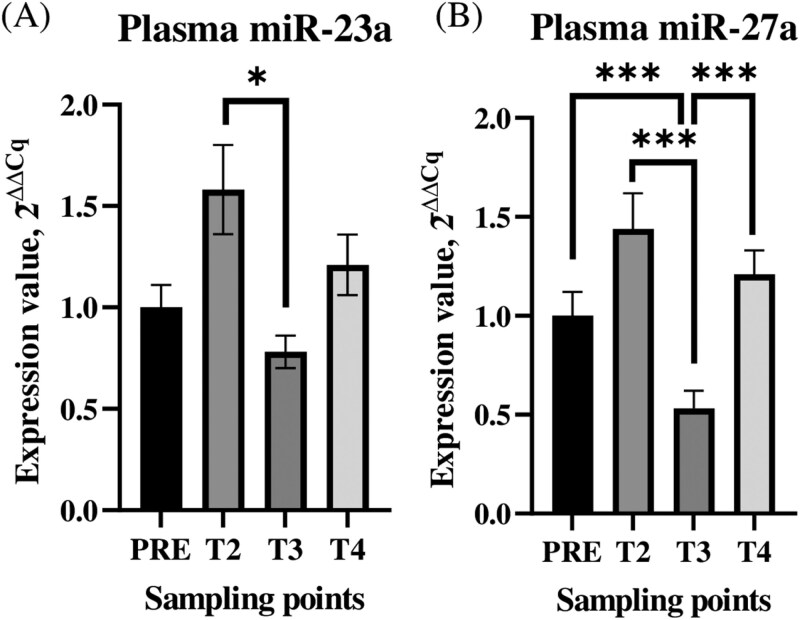
Statistically significant miRNAs in plasma. Statistically significant miRNA expression profiles in the plasma of Rasa Aragonesa lambs at 5 sampling points (PRE: average of T0 and T1). For clarity, miRNAs expression profiles are expressed in 2^−∆∆Cq^. * ~ *P *< 0.05; *** ~ *P *< 0.001.

In plasma, miR-23a was significantly (*P *< 0.05) more expressed after unloading (T2; 3.94 ± 0.67) than it was at T3 (4.74 ± 0.21) (Fig. 5A). In the same matrix, miR-27a was significantly (*P *< 0.001) less expressed in T3 (6.53 ± 0.24) than it was at all the other sampling points (PRE: 5.66 ± 0.36; T2: 5.28 ± 0.54; T4: 5.46 ± 0.37) (Fig. 5B).

### Plasma CK and lactate dehydrogenase concentrations change after transport

**Figure 6.**
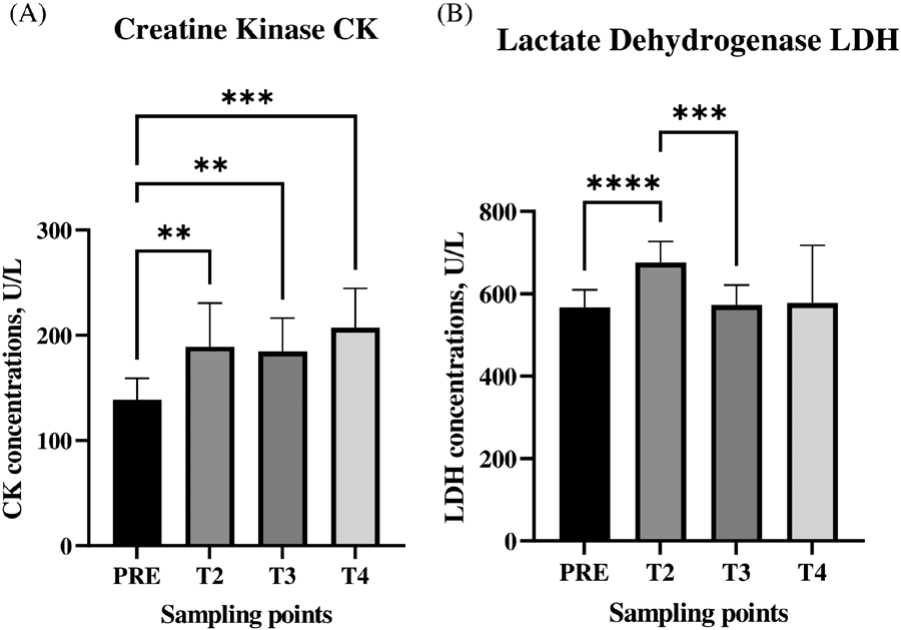
Enzymes evaluation. Average concentrations (±SD) of CK and lactate dehydrogenase (U/L) in Rasa Aragonesa lambs at 5 sampling points (PRE: average of T0 and T1). ** ~ *P *< 0.01; *** ~ *P *< 0.001; **** ~ *P *< 0.0001.

Significant differences in CK and LDH concentrations were demonstrated by comparing the mean of PRE (T0 and T1) transport time points with the no-mediated POST concentrations to better appreciate the trend of enzymes concentration following the main stressful event. CK concentrations in the plasma of lambs increased throughout the experiment. CK concentrations were significantly (*P *< 0.01) lower at the PRE-sampling (138.71 ± 19.72 U/L) than they were at the other 3 sampling times (T2: 189.14 ± 39.95 U/L; T3: 184.36 ± 30.85 U/L; T4: 207.21 ± 36.04 U/L) (Fig. 6A). Similarly, concentrations of plasmatic LDH were significantly higher after unloading (T2: 675.64 ± 49.73 U/L) than they were at the PRE (566.93 ± 41.06 U/L) (*P *< 0.0001) and T3 (572.64 ± 46.35 U/L) (*P *< 0.001) sampling times (Fig 6B).

### Correlations between miRNA expression and saliva cortisol, CK, and lactate dehydrogenase

Correlations between miRNA expression profiles and cortisol, CK and LDH concentrations were calculated (Table 2). Normalized cortisol trend resulted significantly (*P *< 0.05) negatively correlated with the expressions of miR-17 (*r* = −0.60) and miR-24 (*r* = −0.61) in saliva at the T4 sampling point. CK concentrations in saliva and miR-27a expression were significantly (*P *< 0.05) negatively correlated (*r* = −0.68) at the last sampling point (T4).

**Table 2.** Pearson correlations. Pearson correlations between cortisol, CK, and LDH concentrations and miRNA expressions in Rasa Aragonesa lambs before, during, and after transport (*r* and *P* value). T2: immediately after unloading; T4: 24h after transport

Correlation			*r*	*P*-value	Sampling point
Cortisol	–	Salivary miR-17	−0.60	0.049	T4
Cortisol	–	Salivary miR-24	−0.61	0.046	T4
CK	–	Salivary miR-27a	−0.68	0.045	T4
LDH	–	Plasma miR-23a	−0.76	0.028	T4
LDH	–	Plasma miR-23a	0.76	0.029	T2
LDH	–	Plasma miR-24	0.79	0.019	T2
LDH	–	++Plasma miR-27a	0.79	0.019	T2

Abbreviations: miR, microRNA; CK, creatine kinase; LDH, lactate dehydrogenase.

The concentrations of LDH in plasma were significantly (*P *= 0.028) negatively correlated with the expression of miR-23a (*r* = −0.76) at the T4 sampling point. Furthermore, LDH in plasma was positively correlated with the expression of miR-23a (*P* = 0.029, *r* = 0.76), miR-24 (*P *= 0.019, *r* = 0.79) and miR-27a (*P* = 0.019, *r* = 0.79) immediately after the unloading (T2).

## Discussion

The acute-stress response is an evolutionary adaptation for facing unpredictable environmental challenges that activate mostly the SAM and HPA axes (Moberg, 2000; Sapolsky, 2002; Kaltas and Chrousos, 2007). The main physiological effects include changes in metabolic rate, cardiac function, peripheral circulation, respiration, and energy availability (Chu et al., 2024). In that context, analyses of HR and BT provide an accurate assessment of the functional regulatory characteristics of the autonomic nervous system (Sztajel, 2004; Madden and Morrison, 2019; Thomas et al., 2019). In addition, cortisol secretion is the basis of the involvement of the HPA axis (Möstl and Palme, 2002). In our study, the bio-loggers provided accurate records of externally induced alterations in animal homeostasis, which was very useful because it allowed us to identify the exact steps in the transport process at which the adaptation response was activated in the lambs, and provided the stages at which evaluate potential circulating biomarkers such as miRNAs.

The physiological variables measured in our study included those known as indicators of a stressful environment for sheep. In literature, measurements of HR, plasma cortisol, glucose and CK have shown that it is the initial stages of transport that are most stressful to sheep (Knowles et al., 1995; Vogel et al., 2024). In our study, due to methodological limitations, we could not sample the animals at the initial stages of transport in particular immediately after the loading, but interesting results emerged, especially after the end of the journey.

A few previous studies measured both HR and BT in sheep through the use of bio-loggers (Abecia et al., 2021, 2025). In Abecia et al. (2025), it was demonstrated that the management system had a direct impact on the subcutaneous BT and HR of the ewes, whose circadian rhythms were influenced by the timing of concentrate feeding. Indeed, BT reached the lowest point just before feeding and began to rise afterward, peaking immediately post-feeding (Abecia et al., 2025). The waiting of feeding time could fall among activities that Smirnov and Kiyatkin (2008) described as “anticipatory” metabolic activation, thus providing a crucial advantage for the organism’s adaptation to potential energetic demands inducing a rapid subcutaneous temperature decrease (Smirnov and Kiyatkin, 2008). Pascual-Alonso and colleagues (2017), using a data loggers monitored the internal vaginal temperature every 1 min (our same frame of collection) in sheep during different trials of transport showing a consistent increase in internal BT of the transported ewes until a turning point between the end of the journey and unloading. Subsequently, until 24h after transport, the ewes recovered their initial values. In our study, the elevated HR that was associated with a reduction in subcutaneous BT during the unloading procedure that the authors can hypnotized related to peripheral vasoconstriction induced by the activation of the SAM axis (Smirnov and Kiyatkin, 2008; Kiyatkin, 2021). In the end, salivary cortisol concentration peak a few minutes after the changes in HR and BT were registered (Fig. 4). After this, we can’t exclude that, in lambs, elevated cortisol levels may indicate a response to emotional stress triggered by the environment stimuli, rather than being a direct consequence of transport-related stress (Napolitano et al., 1995; Bergamasco et al., 2005).

As in the De la Fuente (2010) and Wickham’s (2012) studies, in our study, the patterns in HR and BT, which were collected with precision by the bio-loggers, indicated that also loading is one of the main stressful stages for the lambs, and much more influential than was the time spent in transport on the lorry. In this context, the lorry used for transport was designed and suitable, thus allowing a passive ventilation with the passage of air due to movement. During transport, the ventilated air removes some metabolic heat, leading to direct convective cooling (Cockram, 2024). Moreover, high temperatures during transport stimulate evaporative heat loss in the animals by panting and sweating (Caulfield et al., 2014). In addition, our study detected the same physiological response during the unloading procedure, i.e., salivary cortisol levels were elevated significantly. Wickham et al. (2012) report that the stress response to transport (increased HR and alerting behaviors) is more frequent in animals not accustomed to this procedure, compared with animals with previous experience. For this reason, the adaptation response of considered lambs, not used to transport procedure, could be amplified or more evident.

Knowledge of the timing of those adaptative changes allowed us to identify the c-miRNAs that are involved at these specific moments, in which we know that the activation of HPA and SAM axes takes place. MiRNAs are a class of regulatory RNAs that have pleiotropic effects on multiple pathways in a tissue-specific manner and interest in the importance of these molecules as biomarkers in assessing animal welfare during management procedures that are stressful. Furthermore, miRNAs play important roles in responses to stress (Manenti et al., 2023; Podgórska et al., 2024), including those associated with transport. The distal signaling mechanism of cell-free miRNAs within peripheral body fluids such as saliva and plasma is not well understood, and in sheep is particularly lacking.

In our study, out of 17 miRNAs (miR-17, -19b, -23a, -24, -27a, -143 and -191) tested, 7 were found to be reliably detectable in the blood and saliva of lambs. Furthermore, our study detected dynamic changes in the levels of miR-17, miR-23, miR-24, and miR-27a, throughout the transport simulation and subsequent body restoration. In other studies, those miRNAs have been shown to be associated with the body’s ability to adapt to physical and psychological stress (Jin et al., 2016; Podgórska et al., 2024). In at least 1 of the 2 matrices, expressions of those c-miRNAs differed significantly between the transport procedures and both the PRE (T0: 24h before and T1: 4h before transport) and the POST (T2: immediately after, T3: 4h after and T4: 24h after transport) samples. Furthermore, statistically significant changes in the expression of miR-23a and miR-27a occurred in both saliva and plasma. Of the 4 miRNAs that indicated stress-induced changes, we focused on miR-17b, which has been shown to be associated with anxiety-like behaviors in mice (McEwen, 2017). Jin and colleagues demonstrated that miR-17 is involved in the regulation of adult hippocampal neurogenesis and mood by influencing the expression of genes in the glucocorticoid pathway, which have been associated with stress-induced anxiety behaviors (Wiegand et al., 2018). Although our study did not assess the lambs at the brain level, we suspect that miR-17 is involved in the complex interactions among mediators, brain region specializations and are common molecules that are associated with the stress response.

In our study, miR-23, -24, and -27 were the other miRNAs that were particularly noteworthy. Several studies have been found their highly expression in skeletal muscle and their involvement in muscle physiology or rearrangement (Miretti et al., 2013; Hernandez-Torres et al., 2014; Tewari et al., 2021). In an *in vivo* study, Lee and colleagues (2019) in double-knockout mice muscle-lacking for miR-23-27-24 clusters confirmed they role with different effects on skeletal muscle development and endurance exercise-induced muscle recovery. Other studies have reported down-regulation of miR-23 in mice skeletal muscle after an acute bout of endurance exercise, but no published studies have investigated this miRNA at the circulating level (Safdar et al., 2009). Previously, we found that miR-27a was expressed in the saliva samples of sheep in the adaptation response in breeding context (Manenti et al., 2023). Others have reported that serum miR-27a is strongly correlated with cognitive impairment in humans (Kondo et al., 2019), and is involved in processes that are important to the response to exercise (Tonevitsky et al., 2013).

Experiments have shown that the regulatory role of miR-24 in the proliferation and differentiation of myoblasts (Hu et al., 2019; Fan et al., 2024). Although the role of miR-24 in skeletal muscle after exercise and injury is unclear, it seems to be involved in the fibrotic process and its upregulation might facilitate the recovery of injured skeletal muscle (Sun et al., 2018).

Serum CK and LDH concentrations are investigated as indicators of the extent of metabolic adaptation to physical training in skeletal muscles (Brancaccio et al., 2006) and indicators of welfare (Nielsen et al., 2021). Concentrations increase considerably after intensive exercise and at sites of muscle pathology (Hood et al., 1991; Garry and McShane, 2000). In our study, however, the short, intense exercise faced by the lambs at the unloading might have involved an anaerobic cellular effort, which would have increased muscle stress. In addition, the literature reports that the pavement of the vehicle affects the stress of the animals: travel on unpaved terrain, as in this case, is more stressful and requires more muscle effort than those paved (Miranda-de la Lama et al., 2011). In our study, after unloading, all of the lambs had increased levels of those enzymes.

Our findings have provided additional evidence of the association between transportation stress and an increase in CK and LDH concentrations (Galipalli et al., 2004). CK concentrations were significantly higher after transport at all sampling points (T2-T3-T4) than they were before transport (T0 to T1). Studies have demonstrated that CK is involved in energy metabolism in skeletal muscle; particularly, in the mechanisms that regulate glycolysis, mitochondrial respiration, and muscular contraction (Teixeira and Borges, 2012). Serum CK levels might indicate fatigue or overwork in non-athlete humans and in animals that are not accustomed to moving (Fabrega et al., 2002; Totsuka et al., 2002). LDH concentrations are associated with stress and muscle fatigue (weariness) (Broom, 2000). In our study, transportation caused a significant increase in LDH, which occurred after unloading. Similar results have been reported by Bórnez et al. (2009a and Bórnez et al. (2009b)), Minka and Ayo (2013). Expanded muscle destruction caused by muscle stress during transportation might be the cause of the increase in those plasma enzymes (Adenkola et al., 2009). Similar results involving CK and LDH have been reported in horses after they have been ridden. As in our study, elevated levels occurred not only immediately after muscle exercise, but also in trends of these enzymes. Indeed, the day after having been ridden, enzyme levels in the horses were lower than they were immediately after exercise, but higher than the basal level (Giers et al., 2023). A recent study (Su et al., 2024) found increased levels of CK but reduces levels of LDH in lambs that had daily exercise for 90 d. Differences in the amount of exercise to which the animals were subjected might explain the difference in LDH concentrations between that study and ours. In the absence of or at low concentrations of oxygen, such as can occur during a short and intense exercise activity, LDH converts pyruvate into lactic acid (Khan et al., 2020). Probably daily and prolonged exercise increases the production of intracellular energy, which improves aerobic metabolism in muscles (Su et al., 2024).

We found positive correlations between trends in the of expression of miR-23a, miR-24, miR-27a, which are highly expressed in skeletal muscle and involved in muscle physiology (Miretti et al., 2013; Hernandez-Torres et al., 2014; Tewari et al., 2021), and LDH concentration trend in plasma immediately after the unloading of the lambs (T2), which suggests that there might be an interaction between CK and LDH and underscores the possibility of using circulating miRNAs as biomarkers in assessing animal welfare related to muscle fatigue. Furthermore, it suggests that management procedures should be revised to reduce muscle fatigue during loading and unloading, given the lambs the opportunity to perform muscular activity (movement) in the fattening period. In our study, the correlations among the expression trends of miRNAs, cortisol concentrations, and CK and LDH levels at the last sampling point (T4) might indicate a delay in the responses of these parameters in response to the stressful event. Other studies (Diver et al., 2013; Robertson et al., 2013; Sabirzhanov et al., 2014) have identified the relationships between those miRNAs and the molecular markers that we assessed in our study (Wang et al., 2012, 2021), and the correlations that we detected provide new approaches to the assessment of welfare animal during management procedures.

Although much remains to be learned about the adaptation response, research has evidenced the importance of miRNAs in maintaining homeostasis (Sharma et al., 2020). Further studies are required to investigate the role of those miRNAs to better understand their contribution to the ovine stress response; specifically, the interactions between miRNAs and their putative target genes.

The development of technologies that provide continuous monitoring of physiological parameters would contribute to a change in farm management practices, and the resulting information might provide the basis for more tailored procedures for animals of a given species at various physiological moments in their life. Continuous monitoring over long periods might be useful in investigating broader physiological questions such as the timing of homeostatic recovery.

A better understanding of the ability of an animal to cope with stressful events might help in the selection of breeding ewes and rams with the aim of producing offspring that are more capable of coping with farm stress events, which will increase the likelihood of high product quality.

Our study revealed the dynamic nature of ovine plasma and salivary miRNAs during transport-induced stress. In linking the expression trends of those circulating miRNAs with physiological stress indicators (HR and BT) and molecular stress markers such as cortisol, CK, and LDH, this study suggested their potential role in understanding the regulation of the acute-phase response and their use as possible stress biomarkers for livestock.

For future studies, in addition to deepening the involvement of these miRNAs in acute-stress response, potential confounding factors during transportation such as road conditions, fluctuation in vehicle speed or environmental noise, that we didn’t record, could be considered to better interpretate stress indicators and analyze animals’ stress response.

## Data Availability

All data used in this study are included in this published article.

## Abbreviations

ANOVA: analysis of variance
bpm: beats per minute
BT: body temperature
c-miRNA: circulating microRNA
CK: creatine kinase
Cq: quantification cycle
ECG: electrocardiogram
HLT: higher-level threshold
HPA: hypothalamic–pituitary–adrenal
HR: heart rate
LDH: lactate dehydrogenase
LLT: lower-level threshold
miRNA: microRNA
PLF: Precision Livestock Farming
QI: quality index
RH: relative humidity
SAM: sympathetic adrenal medullary
SD: standard deviation
SE: standard error
*T*: temperature
